# Mathematically Gifted Accelerated Students Participating in an Ability Group: A Qualitative Interview Study

**DOI:** 10.3389/fpsyg.2018.01359

**Published:** 2018-07-31

**Authors:** Jørgen Smedsrud

**Affiliations:** Institute for Pedagogy, Department for Education, University of Oslo, Oslo, Norway

**Keywords:** mathematically gifted, high-ability students, acceleration, ability groups, giftedness, education for gifted students

## Abstract

Schools in Norway often emphasize heterogeneous groups in education. The postulated negative effects of homogeneous ability groups on motivation and academic self-concept have long been debated. This study uses semi-structured interviews to investigate how a group of 11 accelerated and ability-grouped high-ability students (gifted) in math have experienced school. All students were interviewed individually. This study explores categories connected to the students’ motivation for the subject, challenges in school, peer and teacher relationships, and academic self-concept. The aim of the study is to investigate whether the school system is able to provide an adequate learning environment for high-ability students, both in ordinary class and in ability groups. The findings show that although some of the needs of high-ability students in Norway are being met, there is much work to be done before an optimal learning environment is established for these students. For example, students do not receive sufficient challenges in math. Furthermore, teachers in the early years of school lack sufficient mathematical knowledge to challenge and support mathematically gifted students, students’ motivation for the subject is lower than expected, and boys’ self-concept seems to be higher than that of girls.

## Introduction

Research indicates that teachers lack the relevant pedagogical skills and specific content skills to challenge highly gifted learners in school (VanTassel-Baska and Stambaugh, 2005). Empirical evidence suggests that the main element in fostering mathematically gifted students is learning opportunities (Nadjafikhah et al., 2012; Hoth et al., 2017). Although grouping students according to their ability in a specific subject has not been a traditional way of challenging high-achieving students in Scandinavia. In the last few years, more attention has been aimed at gifted education and gifted research. The Official Norwegian report (2016) concludes that more research is needed on gifted children and high-achieving students within the Norwegian context as well as educational provisions and teacher competence with regard to this group (Børte et al., 2016). One of the most important factors in developing the potential of gifted students is meeting their academic needs (Winner, 2000; Ziegler and Heller, 2000; Clark and Callow, 2002; Montgomery, 2009). There are currently few local schools in Norway with curricula adapted to this group, few programs with opportunities for acceleration, and few ability groups for gifted adolescents who achieve highly. However, some universities and high schools give high-ability students the opportunity to participate in accelerated programs or ability groups.

This study aims to investigate how high-ability (gifted) students in Norway who participated in a special accelerated math program at a university experienced motivation, relationships with their teachers, peer relationships, and perceived self-concept in math. The students participating in the program followed 10 study points from a mathematical course at the university and were tutored one evening a week over a year. At the end of the semester, the students could choose whether to be examined and receive the study points or to follow the curriculum for their own interest. The qualitative interviews were based on a broad understanding of gifted adolescents grounded in the *three-ring concept of giftedness* and Mönks (1992) multifactor model of giftedness, which builds on Renzulli’s thoughts. This approach facilitates the exploration of both the underlying micro and macro factors that lead to gifted behavior and the realization of high potential.

Micro levels are connected to individual differences, such as personality, motivation, and emotional stability, while macro levels are connected to school, teacher, parents, or peers (or other environmental aspects that might have an influence on individual performance). The combination of the concepts allows us to understand giftedness at both broad individual and environmental levels (Davidson, 2009). Renzulli (2002a) understands gifted behavior as a combination of *above-average ability*, *creativity*, and *task commitment*, whereas Mönks (1992) and Mönks and Mason (2000) include school, family, and peers. Mönks’ model has been extended to include time perspective, planning, and emotional factors (Mönks and Katzko, 2005). Above-average ability, which Renzulli considers the top 15–20% of a given age group, refers to a person’s cognitive ability. In Renzulli’s model, high levels of creativity are associated with originality of thought, and task commitment refers to a person’s special interest or commitment to a subject (Renzulli, 2002b, 2003). In Rensulli’s view, giftedness occurs when all three components described are present. Mönks describes how individual (stress, social, and motivational factors) and environmental (school, peer, and parental factors) also influence gifted behavior. In Mönks’ view, task commitment also includes motivation. The top levels of performance involve the top 5–10% of performance in any given domain (Mönks and Mason, 2000). Because gifted behavior only occurs with sufficient stimuli from the environment, it is interesting to explore both individual and environmental traits for a better understanding of how giftedness develops.

The current study seeks to collect information about the micro and macro levels connected to gifted behavior in one specific domain (mathematics). Macro levels include school, peers, or other environmental factors, whereas micro levels are connected to individual traits, such as motivation, emotions, and task commitment. The levels examined are motivation, peer relationships, relationships with teachers, and experiences connected to accelerated and ability groups. To obtain information about individual traits that lead to performance in one academic area, qualitative interviews can provide interesting information about the underlying individual differences or similarities in gifted students. Furthermore, interviews can provide deeper explanations of how these adolescents experience school in Norway.

### Mathematically Gifted Students

Continuum research on the topic of giftedness shows that no single specific criterion can be used to determine giftedness (Sternberg, 1993; Gagné, 1995, 2000; Morelock, 1996; Wellisch and Brown, 2012). Newer interactional models of giftedness see giftedness as a pattern of cognitive, motivational, and social variables needed for high achievement in one or more domains (Vlahovic-Stetic et al., 1999). Thus, giftedness can be understood as a result of several interacting variables that lead to gifted behavior. Factors connected to the micro and macro levels of individuals are important to understand how the needs of gifted adolescents can be met (Vlahovic-Stetic et al., 1999; Reed, 2004). Sowell et al. (1990) suggest two types of mathematically gifted students. The first type of student is typically able to work with mathematical problems at a level of difficulty well above what is normal for their age. The second type is able to solve mathematically complex problems by employing different thinking processes (Reed, 2004). Mathematically gifted students are often capable of high levels of problem solving and inductive thinking. They display high levels of logical reasoning, high self-efficacy, and intrinsic motivation for the subject (Pajares and Graham, 1999; Sriraman, 2003; Koshy et al., 2009; Leikin, 2014; Leikin et al., 2017). Furthermore, mathematically gifted students are often recognized by their ability to solve complex tasks and engage in mathematical thinking that far exceeds that of their relative age group (Sowell et al., 1990; Reed, 2004). The Study of Mathematically Gifted Youth (SMPY) model of mathematically giftedness was developed to identify mathematically gifted students and to help them develop their full potential (Brody and Stanley, 2005; Lubinski and Benbow, 2006; Brody, 2009). In the SMPY model, mathematically gifted students are those who reason exceptionally well in mathematics. The students are identified using the Scholastic Aptitude Test (SAT-M), which consists of 60 multiple-choice tasks that make it possible to discriminate among students who would score high on in-grade-level tests and those who understand mathematics well above their given grade (Lubinski and Benbow, 2000, 2006). Several longitudinal studies of mathematical giftedness suggest that personality traits such as motivation, individual stress factors, interests, and emotional stability seem to play important roles in developing exceptional talent in mathematics (Lubinski and Benbow, 2006). Thus, the individual traits are not guided by a specific definition of giftedness; rather, they serve as the baseline for identifying mathematically gifted students.

### Motivation in Gifted Students

Motivation, self-efficacy, individual stress levels, and academic self-belief are all important factors leading to performance across different levels of intellectual ability (Bandura and Schunk, 1981; Zimmerman, 2000; Pajares, 2003). For gifted students, appropriate challenges in school have a large influence on motivation (Winner, 2000; Phillips and Lindsay, 2006). According to self-determination theory, motivation is connected to intentions, energy, direction, persistence, and endurance (Ryan and Deci, 2000). To develop high motivation, students need stability, psychosocial support, and challenges at their cognitive level. Settings that do not meet these standards can hinder students’ motivation (Ryan and Deci, 2000). Furthermore, to effectively nurture self-efficacy and academic self-belief in gifted adolescents, it is essential to let them engage in meaningful learning situations; otherwise, they are at risk of becoming underachievers (Colangelo et al., 1993; McCoach and Siegle, 2003).

### Academic Self-Concept and Ability Groups

One way of fulfilling some of the needs of gifted students is the use of ability groups or acceleration. Some studies suggest a negative effect of ability groups or acceleration on motivation and academic self-concept. This phenomenon is known as the “*big-fish-little-pond effect*” (Marsh et al., 2008; Nagengast and Marsh, 2012). The theory suggests that it is better to be a high-achieving student with average peers than a high-achieving student among other high-achieving students in a high-ability group (Zeidner and Schleyer, 1999; Marsh and Hau, 2003; Preckel et al., 2008a). The research indicated that academic self-concept, anxiety, and stress related to achievement are lower for students participating in a normal classroom than in a high-achieving classroom. In general, self-concept and academic self-concept are associated with adolescents’ ability to self-regulate and with their individual happiness, self-esteem, and emotional and cognitive outcomes. Cognitive outcomes are typically measured as academic achievements (Shields, 2002; Preckel and Brüll, 2010; Marsh and Martin, 2011). Nevertheless, academically gifted students are typically found to have a higher academic self-concept than average students, which occurs even after participating in ability groups (McCoach and Siegle, 2003). Furthermore, empirical evidence suggests that academically gifted students can experience a decrease in academic self-concept at the beginning of their participation in special programs but that they eventually experience an increase (Dai and Rinn, 2008; Preckel et al., 2010).

### Acceleration and Ability Groups for Gifted Students

Academic acceleration in school refers to opportunities that allow students to move through the school system or curriculum more quickly than the standard pace. Acceleration can take the form of grade skipping, accelerated groups, and compressed curricula (Steenbergen-Hu et al., 2016). In general, previous research has assumed that academically gifted or high-achieving students can experience social and emotional harm from acceleration (Boaler, 2008, 2014). It is suggested that countries that do not promote ability groups but rather allow all their students to follow the same curriculum within a heterogeneous context achieve the best results on PISA (Boaler, 2008, 2014). For example, Finland has received considerable attention for its PISA results and has chosen to group students heterogeneously (Sahlberg, 2014). Research from Britain suggests that ability grouping has no positive effects on children’s mathematical performance and, for some children, even has a detrimental effect (Nunes et al., 2009; Hattie, 2012). However, a meta-study suggests that negative social and emotional problems are an exception (Steenbergen-Hu et al., 2016). Further, the ways in which researchers and schools define ability groups varies greatly (Boaler et al., 2000; Steenbergen-Hu et al., 2016). Empirical evidence suggests that heterogeneous groups favor low-achieving students’ more than gifted students (Fuligni et al., 1995; Linchevski and Kutscher, 1998; Nunes et al., 2009; Boaler, 2014). Zevenbergen (2003) found that high-ability students grouped by their ability were far more positive about their learning environment and had higher attitudes toward mathematics compared with students with lower mathematical ability participating in lower ability groups. Gifted youth who participate in accelerated programs can experience positive impact on their long-term achievement, social and emotional well-being, and enhanced learning compared with their non-accelerated counterparts (Steenbergen-Hu and Moon, 2011; Steenbergen-Hu et al., 2016). Even more important, the assumption that acceleration carries the risk that individuals develop social and emotional issues does not seem to be driven by empirical evidence (Colangelo et al., 2004). However, academic acceleration is not always the right answer for every academically gifted student. Some might not want to participate in an accelerated class or do not wish to change their environment. Nevertheless, the option should be available and should be evaluated as equivalent to regular tutoring (Steenbergen-Hu and Moon, 2011).

### Aims of the Study

The aim of this study was to explore how mathematically gifted students participating in a special ability group at a university in Norway experience their school situation. The understanding of giftedness in this study was guided by the three-ring concept of giftedness and the multifactor model of giftedness, which have similar categories. Other discussions of interest occurred in the interviews. Because little knowledge exists regarding how this student group experiences Norwegian school, open-ended semi-structured interviews seemed to be the best way to capture their individual experiences. This approach allows information to be gathered about how students experience programs intended to meet their learning needs and whether these programs are sufficient in their aims. The categories in the results section include both macro and micro factors described by the participants in the interviews. They reflect the informants’ general focus during their interviews.

## Materials and Methods

### Participants

In this study, the students participated in the accelerated group in their own free time and by their own free will while simultaneously participating in regular school. The participants ranged in age from 16 to19 years old. All of the students had accelerated by one or more years in mathematics within the Norwegian school system, and they passed math r2 in high school (*r2 is the most difficult level of math in the second year of Norwegian high school*). Their home schools varied greatly; however, all students were located in communities near Oslo. Thirteen high-ability adolescents – nine boys and four girls – chose to sign an agreement to be interviewed. However, two withdrew their participation for a final sample of *N* = 11, three girls and eight boys. The sample reflects participation in the program, which includes significantly fewer girls than boys in general. The sample is sufficient for making cross-case generalizations but is fairly small compared with samples used in some other qualitative studies (Robinson, 2014). The participants all showed high levels of mathematical mastery though their school years, and they achieved well above the norm compared to their age group. However, no intelligence testing was included to further validate their intellectual level. Empirical evidence suggests that high intelligence scores and academic performance correlate (Neisser et al., 1996; Geary et al., 2017; McCoach et al., 2017). This type of selection process is referred to as *purposive* sampling, in which the participants are recruited within a specific context (Robinson, 2014).

### Procedure

To secure volunteer participation, during a lecture at the university, information was provided to all students participating in the program. Some weeks later, letters were given to the respondents with information about the project; anonymity and confidentiality were ensured. The letters were based on the ethical guidelines of the NSD (*Norwegian center for research data*). The students who were willing to participate signed the letter and were later contacted by the researcher for interviews. The participants provided both oral and written consent; the written consent was saved according to the NSD’s guidelines. All of the interview data were gathered in a fairly short time span (within 1 month). The interviews were intended to last about 40 min, but in reality, most of them lasted more than an hour, and some lasted up to 1.5 h. A semi-structured schedule was used with open-ended questions based on theoretical descriptions of mathematically gifted students and information about the context of the program. Because the interviews were open-ended, the categories in the interview guide were broad and addressed school history, experience in accelerated classes, how the students perceived mathematics, if they felt motivated toward mathematics, how they experienced the school system in relation to being talented in Norway and their experience with the ability group. The follow-up questions differed between the interviews. The aim was to not guide the interview subjects in their discussion and explanation. Whenever something interesting was brought to light, they were asked to further elaborate. The categories were drawn from the theoretical frameworks of Mönks and Renzulli, which emphasize both the individual and environmental factors leading to gifted behavior. The categories were so broad to allow the interview subjects to focus on their experiences rather than the researcher narrowing and guiding their answers by following a rigid set of questions. After every interview, field notes were made about unclear questions or observations that could influence the meaning of a specific statement. Two tape recorders were used to ensure that all statements were clear and to ensure that a backup was available if any technical issues arose. The transcription process was completed in two stages. In the first stage, one person transcribed all the interviews in Microsoft Word. In the second stage, the same person read through the interviews and corrected any misunderstandings that could have occurred during the interview by also listening to the tape. All of the interviews were conducted and transcribed in Norwegian. The end results were presented in English, which represent a danger of “loss of meaning” (Hammersley, 2010). The problem of meaning affects all types of interviews, and translating from one language to another represents a special issue. To compensate for this problem, metaphors that were not meaningful in English and local idioms were not represented in the citations. To ensure accuracy, the data were checked with the interviews (audio file) several times to improve understanding of the subjects’ meaning (Dalen, 2011).

### Analysis

Castleberry (2012) NVivo version 11^TM^ was used to further analyze the material. Thematic analysis is one of the broadest and most frequently used methods of analyzing qualitative data in educational science (Braun and Clarke, 2006; Kvale and Brinkmann, 2009). By first exploring the material for themes other than those already presented in the interview guide, the study was open to the development of new information and new ways of understanding the concept at hand. Interesting new discussion points, such as *lack of challenges in school* and *the system fails the students*, emerged as a result of the focuses of the informants in the interviews. The analysis was theory-driven (as structural interviews often are). Triangulation is a strategy that is typically used to improve the validity and reliability of qualitative research (Maxwell, 1992; Golafshani, 2003). The triangulation process in this study mainly proceeded from context to informant to theory. The theories represented in the interview guide (e.g., the categories’ micro/macro levels) were understood in line with the informants’ statements and then considered in a broader theoretical framework of gifted education and mathematically gifted students. Triangulation also involves questioning findings that might contradict previously established models or theoretical frameworks. This process was used throughout the entire analytic process. A triangulation process can enhance the validation of the eventual findings and eliminate misinterpretations of the transcribed material. However, a weakness of this research is that one person translated and analyzed the interviews. Thus, the research is guided by that individual’s personal theory and understanding of the participants in the study.

## Results

### Motivation and Interest in the Subject

Intrinsic motivation is often emphasized as an important factor in students’ engagement and performance in academic subjects (Preckel et al., 2008b; Klapp, 2017; Liu and Hou, 2017). Intrinsic motivation is also seen as one of the hallmarks of high-achieving gifted students (Al-Dhamit and Kreishan, 2016; Leroy and Bressoux, 2016). In this study, the students did not perceive math as a subject in which they were more motivated than other subjects, even though they had skipped grades – and, in some cases, several years – in mathematics. First, they often described a lack of opportunities for acceleration or enrichment activities in other subjects as a result of choosing math as their subject. Second, many described math as a subject they understood easily; therefore, they used little effort when studying or preparing for a test.

“It is not really math I like the best of the subjects, I like chemistry and physics; however, we did not receive any tutoring in those subjects during elementary school or junior high school. We didn’t get a chance to accelerate in those subjects either, so only math was left….” (Boy).

Norway has no tradition of enrichment activities for high-ability students outside of school in science or other subjects. Few of the participants were participating in any activities connected to their talent outside of school. The informants described mathematical understanding as something different from understanding within other subjects. They often described mathematical knowledge as connected not necessarily to motivation but rather to understanding, and math was the only subject in which teachers could easily identify their ability. Interestingly, many of the students identified the reason for their understanding of math as outside their own control, describing themselves as lucky “*to be born with a brain to understand math*.” Often, they found that they thought differently about math when they compared themselves to other students.

“It’s not really allowed to say; however, I feel that when I talk to others, they have a totally different understanding of mathematics than me… For me, a certain answer just makes sense... I feel like I have a different approach and understanding, but if someone asks, I can’t really describe why; my brain just works in that way…” (Boy).

Because they interpreted mathematics in a way that differed from other students, they often did not feel they could discuss mathematics with other students and therefore had to stimulate their interest in arenas other than school. In this way, most of their motivation was driven from within.

### Mathematical Ability and Academic Self-Concept

A majority of the students described math as something they had a talent for in other ways than their particular interest in the subject. In general, the students needed little time to understand mathematical problems in high school. Furthermore, most felt that there was a general problem with the way math is taught in Norwegian schools. The general approach did not meet their understanding of mathematics. The informants highlighted two different approaches to math: they felt that the understanding of math was under-communicated in school and that teachers focused on memorizing models before a specific test.

“One thing is to use the theory to calculate something. One must focus on understanding the theory; then you can understand why the results are the way they are… The way I understand math is like a language just like in other topics... Especially when I talk to other students, I explain math as a language... A language you can use to explain all other sciences... Therefore, you have to explain and emphasize a more underlying understanding of math in school because understanding math as a language can give you a deeper understanding of physics, chemistry and biology.” (Girl).

In the students’ view, a greater focus on the underlying theoretical perspective of math would be a much better way of teaching the subject in school. When asked why they thought this was a better approach to teaching math, they explained that if you memorize something, you do not really understand the concept. However, if you truly understand a concept, you can apply the understanding to a broad range of problem-solving strategies. “*If you spend more time on understanding the concept, even though some might not understand it, I think it would make math easier in the long run for everyone, even the weaker students*” (Boy).

When comparing themselves to average students, some students felt it was somewhat unfair that they needed to use so little effort before a test or to understand a certain topic. One student felt she had to lie to the other students about how long she actually studied before a test: “*… I can feel guilty if my friends tell me they have worked really hard before a test and I have not. Then I often just say I also have used a lot of time to study before the test. I have friends that work a lot more than me and I get much better grades*” (Girl). The few girls in this study seemed to be more focused on what their peers might think about their performance. This statement can be connected emotional support. It seems that several of the participants described a lack of emotional support from the environment, from their teachers, and, in this case, from friends. Thus, they received emotional and social support from the other participants in the ability group – mostly because they did not perceive their participation as competition.

“It was such a good experience. I don’t really care whether or not I receive a good grade on the exam now. I met others that thought like me; it was really fulfilling. Of course, I like my other friends also; however, it was so cozy to have them around me in the group. It is nice to feel that math is cool; we are not exactly perceived as cool in ordinary school” (Girl).

For most of them, their most positive experience at the university was related to the perception of their talent as something positive and that they did not need to “hide” their engagement: *With my other friends who are not so good at mathematics, I cannot just talk out loud about how good I am. They would probably think I was bragging about my abilities* (Girl). Their academic self-concept seemed high in general. However, in contrast to what one might expect, the statement above can be understood as an increase in academic self-concept after participating in an ability group. In their view, their mathematical talent was normalized by mirroring her interests with other like-minded students.

### Are High-Ability Students Sufficiently Challenged?

In the interviews, all of the participants were asked how they felt their academic needs were met through school. Most of the students reported receiving far too few challenges in primary school and middle school. All of the informants had skipped one or two grades in math during middle school or later, but none of the students had been invited to skip grades or to participate in another classroom while in primary school. Some of the students had been allowed to work alone with a book used by higher grades, but often with little tutoring from the teacher in class. The most significant issue was the lack of individualized challenges and how this affected the students’ learning. As the main problem, they described feeling a lack of motivation and boredom because of repetition and too few challenges: “*I think it is a good idea not to let those who really have a talent or a genuine interest in a subject just sit there and get frustrated by repetitive tasks, which they don’t need to do”* (Boy). In some cases, the students even read books on other topics or focused on other tasks during math lectures:

“…As I said, I was sitting and reading a book; it was probably because I did not feel it was necessary to pay any attention to the teacher. It’s like this: if the teachers ask any control questions, you just answer right on the question, and the teacher gets kind of astounded and quiet, and then you just keep sitting there.” (Girl).

When the students were asked what the school or the teacher should have done to better challenge them in math, two discussion points emerged from the material. The informants felt that subjects in school were taught at a slow pace and had little depth. The fact that schools often emphasize group work also stands out as negative for the high-ability students. *“Slow is probably a good word for describing it. You use a lot of time to learn very little and to understand very little”* (Girl). They did not feel the teacher was able to explain the depth of mathematical knowledge or that they individually received a broad explanation of the subject through participation in the regular classroom. Some explained the lack of challenges as a result of the lack of teacher competence. In the students’ view, it should be easy to individualize the speed at which the teachers move through a subject, especially compared with individualized one-on-one tutoring.

“When you are really good in a subject, you feel like you only learn about the surface of the subject. It is way too slow... Slow on the surface. You are in a way just rowing in a canoe on the surface without any oars, and you need to use only your hands to get forward. It is too slow.” (Girl).

It is evident that in the students’ experience, there is too little acceleration and a dearth of customized learning opportunities. The Norwegian system is not properly structured to attend to the needs of accelerated students because they fall between school levels or teachers. All of the students were very satisfied with acceleration as a way of ensuring that they learned faster and more deeply. However, in many cases, the system did not function properly. Therefore, the students had to make choices between subjects they liked, or they received challenges for a period of time that then stopped.

“In my case, I have skipped two years of math. However, there is nothing to do next year because I’ve skipped two grades. Maybe I could go on another course at the university, but during daytime I need to be at my regular school. I can’t just drop out of regular school to go on a course, and it probably would not be customized to fit my needs” (Boy).

In general, the students were very satisfied with the opportunity to accelerate in math. For some, it was life changing to be recognized for their abilities and to have the opportunity to learn at their own pace. Several commented that if their needs had not been properly recognized at some point or if they had not at least received some opportunity to enrich their understanding of certain subjects, they probably would have suffered throughout their entire school career. It is interesting to note that most of the students describe it as important to be “*allowed*” to learn at their individual pace; in Norway, it is the law that students have the right to receive tutoring customized to meet their individual needs.

It is extremely important that you actually are allowed to work at your level. That you get to experience the joy of challenges... Of course, it is a joy to be ahead of everyone else and work with others who enjoy the subject as much as yourself. If I had to continue working on the same level as everyone else, I would probably continue to experience math as boring, so, in a way, it has changed my entire life that I have been allowed to be ahead of everyone else (Boy).

Some students felt a certain level of anxiety related to time pressure because they participated in the accelerated group in addition to everything else. Other students enjoyed working at their own pace and learning more within the subject. At the same time, they felt the advantage of being accelerated was overrated because they still had to wait to start at the university.

### Teacher and Peer Relationships

In their ability to develop positive personal relationships with high-ability students, most teachers were rated highly by the informants, with the exception of some individual teachers. The students generally described their *social relationship with their teacher* as positive. “*I feel I’ve had a good relationship with my teachers in general; however, I felt that math was pretty easy early on in school”* (Boy). Some students had experienced negative feedback or negative attitudes toward their personality or negative reactions when they asked difficult questions in class. They did not necessarily feel the need for a teacher to instruct them, and it was more important to them that the teacher not hinder their progress than support them academically. However, with regard to academic knowledge and whether they felt supported by – or needed academic support from – the teacher, most students felt the teacher-student relationship to be somewhat overrated. The students’ little need for academic support in the classroom could be explained by the fact that they were given few individual challenges and that most of the challenges they did receive were things they already understood on their own.

I remember that a friend and I were taken out of class and sat in a room and were given some harder tasks, but without any explanation or introduction to them. And then it was suddenly too difficult, so that didn’t really help much. Then we had to participate in the regular classroom again, where I already knew everything. The teacher probably thought, “Oh, so they were not that smart after all.” (Boy).

When describing whether academic support or their intrapersonal relationship with the teacher was more important to them, all the students agreed that academic progress was important to their thriving at school. They all explained that the teacher was important for personal support and understanding. However, in their earlier stages of schooling, they had not received any specific academic challenges. In some cases, they had served as an assistant teacher or were asked by the teacher to explain a difficult concept. Furthermore, their subjective experience with teachers in school reflected the teachers’ poor understanding of the academic needs of high-ability students. However, individual teachers made a significant difference in helping the students explore their talent and in introducing them to new and exciting subjects. From the participants’ perspective, the most important teacher characteristics for learning were dedication and deep knowledge of mathematics. Nevertheless, there was a general consensus among the students that the largest difference between the university and regular school was that at the university, they actually needed the teacher if they wanted to understand the concept at hand.

*A positive relationship with peers* was important to all informants. All the students experienced the opportunity to participate in acceleration programs as positive in general, and they were excited to meet others who were even better than they were at mathematics. All of them described their interpersonal skills as sufficient and saw no negative associations between their talent and developing friendships; rather, they perceived their talent as something positive. Furthermore, their attitude toward the other participating students was positive, and many explained that they were socially compatible in another way compared with their average peers. For example, they shared a common understanding of and interest in mathematics, which they had not previously experienced in the same way with other peers, even though they had previously participated in acceleration groups. However, most of them had one or two friends who were also talented in mathematics. The fact that they shared this interest meant a lot to them: *Without my two friends, I think it would have been very difficult without them, even though my family supports me* (Boy). Others felt the fulfillment of academic challenges was more important than friends or friendships: *From my perspective, I would much rather have sufficient challenges academically than participate in a classroom or… with people at my age* (Boy). The last quote underlines the importance of academic challenges if high-ability students are to thrive and feel that their talent is accepted. Furthermore, in this category, it is also clear that the need for social relations differs among the students. Although contact with other academically talented students is seen as important for gifted and high-ability students, some expressed the importance of heterogeneity in their friendships.

## Discussion

The general gender distribution of the program was interesting. Significantly, more boys than girls participated in the group, which does not reflect newer research showing that in Norway, girls tend to do better in math than boys. There is little information about differences in interest in math between boys and girls in Norway. Although several trends can be identified in the way boys and girls describe their experiences, there is a lack of sufficient data to draw conclusions about these trends in this particular study. Halpern et al.’s (2007) study found gender differences in the interest in science and mathematics that favored boys at the highest levels of cognitive ability. This study might explain why there are so few girls in this particular group. A study by Hyde et al. (2008) described how gender differences in mathematics emerge in high school and college, which is the same age group as the informants in this study. It might be that the gender stereotypes are stronger in this age group. Furthermore, *the greater man variability hypothesis* is one explanation for why boys tend to have a greater variance in performance and test scores than girls do (Hyde and Mertz, 2009; Else-Quest et al., 2010). In general, boys seem to be overrepresented at the very top or bottom of any topic, even if there is no average gender difference (Hyde, 2005). In some cases, it might be that girls are more drawn to subjects other than science and mathematics. Nevertheless, the few girls in the study described more stress connected to high performance situations, especially in test situations. This tendency can be interpreted as the girls displaying a lower academic self-concept. Van Boxtel and Mönks (1992) related inner stress in performance situations to lower academic self-concept, whereas the stress related to tests can affect their general academic performance and academic self-concept. Moreover, it can also be understood in the context of math anxiety; girls tend to show higher anxiety when performing mathematical tasks than boys do (Devine et al., 2012). Wu et al. (2012) found that math anxiety was present in both high-achieving and low-achieving students, although the reported anxiety was strongest in the low-achieving students. Dowker et al. (2016) related math anxiety to expected performance, and these students already perform at a high level, thus also displaying a high level of expectations regarding their own performance. It might be that the interest and enjoyment in the subject is overridden in specific test situations. The students were interviewed only about mathematical knowledge compared with self-concept; it is possible that their descriptions could be different for other subjects.

All the students participating in this study showed above-average capability in mathematics. Whether the students were creative was more difficult to detect through the interviews. However, most of the participants described mathematics as a creative subject. They considered math as a tool for understanding other subjects in science, and a deep assimilation of math and understanding its applications can be viewed as creative. Furthermore, most of the participants showed high levels of task commitment when working with mathematical problems; they had a genuine interest in searching for an answer and did not describe the process as exhausting, but enjoyable. In contrast, the fact that most of them did not describe themselves as more motivated toward math than other school subjects was interesting. In gifted research, motivation is often described using two categories: first, as a stable personality trait or characteristic and second, as an environmentally induced transitory state (Dai et al., 1998). Beliefs, values, and attitude are all important factors that determine whether gifted individuals achieve in a certain domain (McCoach et al., 2013; Siegle et al., 2017), along with goal orientation and mindset (Dweck, 2012). In this study, the boys in particular showed high levels of goal orientation in math; they found their motivation in earning good grades, whereas the girls were more driven by interest for the subject. The high levels of self-assurance in their own capabilities in mathematics displayed by the boys were in line with these descriptions. They were unafraid of failure and enjoyed competition. However, most of the students also described an increase in motivation when they participated in ability groups in school. If motivation is a personality trait, it might explain why the students did not lose their motivation for the subject during the earlier stages of school. Motivation seemed to be stable among the students and appeared to be based on their innate interest in learning. It is possible that the groups of high-ability students who display high levels of performance in their early years but do not display this stable motivation are at a higher risk of becoming underachievers at an early stage in school, which could occur before any acceleration opportunities or ability groups are presented to them. A typical understanding of underachievement is a rather large discrepancy between potential and performance (Dowdall and Colangelo, 1982; Whitmore, 1986; McCoach and Siegle, 2003). If gifted students are not sufficiently challenged, they are at great risk of becoming underachievers, and this can happen in the early stages of school (Clinkenbeard, 2012).

One of the most important factors for motivating gifted students is to provide them with opportunities to learn at their level of pace and based on their interests (Phillips and Lindsay, 2006). Challenges based upon individual levels of understanding are important for maintaining motivation in general (Wallace, 2000). Many underachieving gifted students experience problems with peers, teachers, and self-regulation (Reis and McCoach, 2000; McCoach and Siegle, 2003). In this study, no such problems were described. To determine whether a student is underachieving, specific information about the student’s potential is necessary. The students in this study had never been tested on their abilities. They had only been identified through high performance in math. It might be that if the students had received individual tutoring in their early schooling, they could have perceived their current level of performance. The study consequently ruled out any information about underachievers because the selection for the study only favored those already performing at a high level.

In general, it seems, at least for this group, that the early years of schooling in Norway are the crucial years in which their needs were not met. The classroom setting is homogeneous, and the teacher’s focus is on the general student population. The students felt that much of their learning in the early years was a waste. In high school, the students had fewer negative experiences, and their needs were meet more sufficiently. However, few of the informants felt their needs were met through participation in the regular classroom; rather, acceleration opportunities made a difference for these students. The tendency described above can be explained through international surveys such as TIMMS, which indicates that Norwegian teachers score low on mathematical knowledge (Hoth et al., 2017). A teacher’s mathematical knowledge might affect their ability to provide sufficient challenges and communicate mathematics aimed at highly gifted learners. Nadjafikhah et al. (2012) emphasized that a teacher needs the ability to discuss complex ideas and understand mathematics at a high level to provide support and guide mathematically gifted adolescents in their learning process. Assouline et al. (2011) noted that even though acceleration is a good way of meeting gifted adolescents’ needs, the teacher must be able to guide them through the more challenging curriculum and must have high mathematical understanding to identify gifted students. The teacher must have a broad repertoire of mathematical problem-solving activities and strong pedagogical content knowledge to foster mathematically gifted student’s needs (Goldin, 2017). It is one thing to align the curriculum for gifted students; it is another to foster and recognize mathematical creative thinking. Creative and divergent thinking can be understood as the ability to generate new and numerous ideas in a given field (Preckel et al., 2006). Mathematically gifted students often perceive and process mathematics in a complex way, and the teacher must be able to do the same (Leikin et al., 2017).

Despite the empirical evidence suggesting that gifted adolescents participating in ability groups might experience negative peer competition and lower academic self-concept, in this study, the informants described their experiences as positive, and they experienced competition as something positive and driven from within. This finding indicates that they mostly competed with themselves and did not perceive any negative peer competition. This tendency is in line with research on highly gifted achievers and ability and/or acceleration (Colangelo et al., 2004, 2010). With regard to acceleration in Norway and whether it is a positive experience for high-ability students to be accelerated, there was consensus among the informants. None of the students in the study described acceleration as something negative. Their descriptions of the perceived positive experiences with acceleration are in line with the findings of Hornstra et al. (2017). In their study, high-ability students participating in acceleration programs had generally positive experiences, both academic and social. Furthermore, part-time programs were even more sufficient for high-ability students than full-time programs (Hornstra et al., 2017). They all described their teachers as successful in developing positive relationships with students. However, they questioned their teachers’ lack of mathematical knowledge. Therefore, teachers with high levels of knowledge may be important for high-ability students, and having teachers who are better at providing emotional support may be more important for students with lower performance in math. If other opportunities, such as more enrichment activities in school or in the classroom, were available, they may have felt differently. These students have few other opportunities; therefore, they are likely to take whatever option is available. The negative aspects associated with acceleration and ability groups were connected to the school system and not to the fact that the students had an opportunity to learn at a faster pace. Furthermore, because the system often does not recognize or offer acceleration at a younger age, the participating students might represent an opportunistic and highly motivated and devoted group, whereas many gifted children might have lost their interest and motivation in earlier years.

It might be that we in Norway are too afraid of implementing acceleration and ability groups as a general option for students who perform at high levels. The fact that at least one girl did not feel she could be honest even with her friends about how she perceives and understands mathematics may also be connected to the egalitarian ideology and fear of elitism in Norway. Norway is a country that tends to respect talents and abilities developed through hard work more than those that can be explained by inherent potential. Fear of elitism and egalitarian ideology is strong in Norway and can, in some cases, explain both why we do not support gifted adolescents and why politicians do not discuss ways of meeting these students’ needs in school (Skogen, 2010). The students who were accelerated in mathematics took all of the possible opportunities that they received (i.e., they chose to participate whenever they got the chance), which is more than we can expect from all students in school. Therefore, it is likely that there are large numbers of high-ability students in Norway who do not receive the same opportunities. Coincidences lead to student’s participation in accelerated classes or ability groups in Norway, meaning that individual teachers or geographic affiliations make a major difference with regard to introducing students to acceleration opportunities. Opportunities to learn should not be limited to the students who are located in a certain geographical area or who have the luck of having a teacher that knows about a specific program. Since there is so little history of facilitating special learning opportunities for high-ability students in Norway, it could be that personality traits connected to motivation, self-beliefs, and goal orientation are even more important for these students over time. Therefore, highly gifted students who are at risk of becoming underachievers might have lost their motivation and, in a worst case scenario, might have already dropped out of school due to insufficient challenges. Studies of high-ability students suggest that boredom predicts underachievement in school (Obergriesser and Stoeger, 2015). Boredom can occur at the very beginning of school and as early as kindergarten in some cases (Mooij, 1999; Little, 2012). The students in this study also described boredom. However, their goal orientation and ability to work with math over time seemed to play an important role in protecting them against becoming underachievers.

## Limitations

Several limitations of this study should be addressed. First, the conceptual framework of Renzulli and Mönks is broad in its conceptual understanding of giftedness. Their model does not clearly define gifted behavior or explain important differences between gifted individuals (Kaufman and Sternberg, 2008). In this study, the categories presented in the results section are broad and might not be explicitly connected to the models presented in the theory section. Thus, the models guide the categories by describing environmental and individual aspects in the population at hand and describing key points in the students’ interviews. No single study can be generalized, especially not a qualitative study. The study presents results from a few interviews with students who were high performing in math. The results may have been different with more participants or with interviews with students who did not perform at a high level. It is not possible to replicate this type of interview study. In particular, it is not possible to duplicate studies that have open designs, such as open-ended interviews, because the context and the interviewer affect the results. Open-ended interviews make it possible for the participants to focus on how *they* experience their own life situation. These interviews are limited by the researcher’s ability to engage and ask follow-up questions that take advantage of *moments of interest* in the interview. In this study, higher mathematical knowledge and more conceptual understanding of giftedness by the researcher could have enhanced the quality of the follow-up questions and may have provided more interesting discussion points. The selection criteria in this study were rather limited; although the students were gifted, we should have obtained more information. Specifically, it may have been interesting to obtain intelligence scores to determine whether students were *exceptionally gifted*. If students were exceptionally gifted, other theoretical aspects could have been added to enlighten the findings and guide the interviews. In the study, underachievers were automatically ruled out because the study only recruited participants through performance mathematics. The results might have been different if underachieving gifted students or gifted students who had not received similar opportunities were interviewed. The results of the study only permit speculations about the general population of mathematically gifted adolescents in Norway. Although the current study raises many interesting discussion points, more thorough research is necessary to better understand the situation of gifted adolescents.

## Conclusion

Despite the limitations presented above, some conclusions can be drawn from this study. The most important finding of this study was that all of the students perceived acceleration and ability groups as something positive. These groups should be part of a changing paradigm in which all gifted students are at least given such opportunities. The students did not experience any negative pressure or competition in the accelerated groups. During the first 10 years of schooling in Norway, too little effort is put into meeting these students’ needs; some of them receive more support when they enter high school. The students seemed to display characteristics associated with the three-ring concept of giftedness, and task commitment was seemingly a very important variable for their success throughout school. Although all of the students displayed above-average ability, creativity was more difficult to identify. More studies are needed that examine ways to create learning opportunities for high-ability students in Norway before it can be concluded that acceleration and ability groups are the best way to meet their needs. We should address how we recruit adolescents to these groups to ensure that we include all students who may need extra activities to feel stimulated in school.

Students’ understanding and enjoyment of working with complex and deep problems was not reflected in the way they were treated by their teachers in the early stages of school. Even though the students in this project were high achievers, many wondered whether they could have learned more and done more throughout their school years. It is evident that too little is done for high-ability students in Norway, and acceleration may be one way of meeting some of the needs of gifted students in Norway.

## Ethics Statement

This study was carried out in accordance with the Norwegian ethical guidelines for qualitative interview studies.

## Author Contributions

The author confirms being the sole contributor of this work and approved it for publication.

## Conflict of Interest Statement

The author declares that the research was conducted in the absence of any commercial or financial relationships that could be construed as a potential conflict of interest.
